# Patients’ Baseline Characteristics, but Not Tocilizumab Exposure, Affect Severe Outcomes Onset in Giant Cell Arteritis: A Real-World Study

**DOI:** 10.3390/jcm11113115

**Published:** 2022-05-31

**Authors:** Cyril Dumain, Jonathan Broner, Erik Arnaud, Emmanuel Dewavrin, Jan Holubar, Myriam Fantone, Benoit de Wazières, Simon Parreau, Pierre Fesler, Philippe Guilpain, Camille Roubille, Radjiv Goulabchand

**Affiliations:** 1 Internal Medicine Department, CHU Nîmes, University of Montpellier, 30029 Nîmes, France; cyril.dumain@chu-nimes.fr (C.D.); jonathan.broner@chu-nimes.fr (J.B.); erik.arnaud@chu-nimes.fr (E.A.); jan.holubar@chu-nimes.fr (J.H.); myriam.fantone@chu-nimes.fr (M.F.); 2 Intensive Care Medicine Department, Lapeyronie Hospital, CHU Montpellier, 34090 Montpellier, France; e-dewavrin@chu-montpellier.fr; 3 Department of Internal Medicine and Geriatrics, CHU Nîmes, University of Montpellier, 30029 Nîmes, France; benoit.de.wazieres@chu-nimes.fr; 4 Department of Internal Medicine, Limoges University Hospital Center, 87042 Limoges, France; parreau.simon@mayo.edu; 5 Department of Internal Medicine, Lapeyronie Hospital, CHU Montpellier, 34090 Montpellier, France; p-fesler@chu-montpellier.fr (P.F.); c-roubille@chu-montpellier.fr (C.R.); 6 PhyMedExp, INSERM U1046, CNRS UMR 9214, University of Montpellier, 34295 Montpellier, France; 7 Department of Internal Medicine and Multi-Organic Diseases, St. Eloi Hospital, CHU Montpellier, 34295 Montpellier, France; p-guilpain@chu-montpellier.fr; 8 Institute for Regenerative Medicine & Biotherapy, St. Eloi Hospital, University of Montpellier, INSERM, 34295 Montpellier, France

**Keywords:** giant cell arteritis, glucocorticoids, tocilizumab, overweight, diabetes, infections, cardiovascular events

## Abstract

Objectives: Giant cell arteritis (GCA) is associated with severe outcomes such as infections and cardiovascular diseases. We describe here the impact of GCA patients’ characteristics and treatment exposure on the occurrence of severe outcomes. Methods: Data were collected retrospectively from real-world GCA patients with a minimum of six-months follow-up. We recorded severe outcomes and treatment exposure. In the survival analysis, we studied the predictive factors of severe outcomes occurrence, including treatment exposure (major glucocorticoids (GCs) exposure (>10 g of the cumulative dose) and tocilizumab (TCZ) exposure), as time-dependent covariates. Results: Among the 77 included patients, 26% were overweight (BMI ≥ 25 kg/m^2^). The mean cumulative dose of GCs was 7977 ± 4585 mg, 18 patients (23%) had a major GCs exposure, and 40 (52%) received TCZ. Over the 48-month mean follow-up period, 114 severe outcomes occurred in 77% of the patients: infections—29%, cardiovascular diseases—18%, hypertension—15%, fractural osteoporosis—8%, and deaths—6%. Baseline diabetes and overweight were predictive factors of severe outcomes onset (HR, 2.41 [1.05–5.55], *p* = 0.039; HR, 2.08 [1.14–3.81], *p* = 0.018, respectively) independently of age, sex, hypertension, and treatment exposure. Conclusion: Diabetic and overweight GCA patients constitute an at-risk group requiring tailored treatment, including vaccination. The effect of TCZ exposure on the reduction of severe outcomes was not proved here.

## 1. Introduction

Giant cell arteritis (GCA) is a large-vessel vasculitis, which mainly affects patients over 70 years old (73% are ≥70 years old [1]), especially women. The disease may affect vision, but cardiovascular events and infections have become a common cause of morbidity and mortality in this aging population [2,3], a fact potentially exacerbated by glucocorticoids (GCs) use [4,5,6,7]. Indeed, the classic standard of care for GCA is based on long-term GCs therapy, which is associated with common severe outcomes such as cardiovascular events and infections [8,9]. Tocilizumab (TCZ), an anti-interleukin 6 receptor-targeting antibody, can be used in case of GCs resistance, GCs intolerance and GCs dependence [10]. Its safety and efficacy have been proven in controlled studies, but few data have been published in real-world patients [11,12,13,14]. It remains unclear whether TCZ, as a GC-sparing agent, reduces severe outcomes such as GC-related adverse events [15]. Limited data exist on the impact of patients’ characteristics at diagnosis of GCA (especially cardiovascular risk factors) and on the impact of treatment exposure (including TCZ use) on the occurrence of severe outcomes in real-world patients.

We aimed to describe the incidence of severe events (including GC-related adverse events) in a “real-world” population of GCA patients, to identify predictive factors among patients’ characteristics at diagnosis of GCA, and treatment exposures (including TCZ exposure, cumulative GC dose).

## 2. Materials and Methods

### 2.1. Study Population

In this single-center, retrospective, observational study, we studied patients with GCA followed up in an internal medicine department at a tertiary hospital in France. Data were collected from patients admitted between January 2011 and December 2020, with a minimum follow-up period of six months. The diagnosis was made by experts using a combination of clinical and paraclinical criteria, in keeping with the American College of Rheumatology (ACR) and GiACTA study criteria [16,17]. The patients with missing data and questionable diagnoses were excluded. The patients were informed with a non-opposition letter. Our study was approved by the local institutional review board (No. 19.10.07).

### 2.2. Outcomes

The primary outcome was the occurrence of severe outcomes during follow-up, including myocardial infarction, stroke, peripheral arterial disease, aneurysm and/or dissection of the aorta, new-onset or worsening (treatment escalation) of hypertension and diabetes, hospitalization for heart failure, chronic kidney failure (estimated glomerular filtration rate < 60 mL/min), all infections, fractural osteoporosis, cataract, gastrointestinal hemorrhage, severe neuropsychiatric symptoms, and all-cause mortality, along with their dates of occurrence. Those outcomes combined classical GC-related adverse effects and other typical causes of morbidity in this frail aging population that can also have a significant impact on the loss of autonomy. For the GCA patients lost to follow-up, we updated our records after a call to general practitioners and/or patients, focusing on the vital status and ambulatory events, such as non-severe infections.

The secondary outcome was to describe potential predictive factors of the first severe outcome from the baseline patients’ characteristics (including cardiovascular factors and GCA characteristics) and treatment exposure, i.e., cumulative GCs exposure (where >10 g was classed as major exposure) and TCZ exposure.

### 2.3. Data Collection

Data taken from the computerized medical records were baseline clinical data, laboratory tests, pathological (temporal artery biopsy [TAB], when available) and radiological characteristics, including medical history, especially regarding cardiovascular events and cardiovascular risk factors. At baseline, constitutional symptoms (fever, asthenia, anorexia, or weight loss), cephalic symptoms (headache, scalp tenderness, or jaw claudication), ophthalmological symptoms (transient or permanent vision loss allegedly due to GCA), cough, as well as polymyalgia rheumatica symptoms were recorded. We also recorded baseline overweight (body mass index (BMI) ≥ 25 kg/m^2^), which may be considered as an early frailty marker among older patients [18,19].

GCA treatment strategy was collected. Osteoporosis prevention was defined by the prescription of calcium, vitamin D, and bisphosphonates. Vaccine management was defined by prescription/update of influenza or pneumococcal vaccines around the time of GCA diagnosis.

Relapse was defined by the recurrence of the initial symptoms of GCA and/or an increase in the CRP level (>5 mg/L) without any other explanation than relapse. Remission was defined by the absence of clinical signs and biological inflammatory syndrome (CRP ≤ 5 mg/L) [20,21]. Our data collection met the EULAR recommendations about research on GCA [22].

### 2.4. GC and TCZ Use

GC doses were calculated as prednisone-equivalent based on the accepted standards. We calculated the mean GCs dose per day (mg) for the treatment duration, and the cumulative GCs dose. The mean cumulative GCs dose in GCA patients was previously reported to be around 9 g (prednisone-equivalent) [6,7,23,24]: therefore, we defined a major exposure to GCs as a cumulative dose > 10 g (prednisone-equivalent) [23].

The reason for TCZ prescription was collected (i) either within the first 3 months of treatment (early-treated patients) because of the rapid onset of GC-related adverse events, oitially refractory disease with remittent symptoms, or persistent systemic inflammation despite appropriate GC therapy (ii) or after conventional therapy with relapsing disease or severe GC-related adverse events. TCZ safety events were also collected.

### 2.5. Statistical Analysis

Descriptive statistics are presented as the means (±SD) or proportions (%) where appropriate. Comparisons were performed using the Wilcoxon–Mann–Whitney rank sum test or Student’s test for quantitative variables and the chi^2^ test or Fisher test for qualitative variables when appropriate.

Using the Cox proportional hazards models in the survival analysis, we studied the comparative incidence of severe outcome according to the baseline characteristics. In case of multiple events occurring in a single patient, only the first event was integrated in the survival analysis. The duration of the follow-up was calculated as the interval between diagnosis and the date of the first detected event, or until the end of the follow-up. To assess whether the incidence of these outcomes was mediated by treatment exposure, we performed an additional model involving TCZ exposure and major GCs exposure as time-dependent covariates. The analysis was performed using the available data (no imputation of missing data). Curves depicting survival analyses do not involve adjustment factors. All the statistical analyses were calculated using RStudio^®^ (v 1.4.1717, RStudio: Integrated Development for R, PBC, Boston, MA, USA).

## 3. Results

### 3.1. Patients’ Baseline Characteristics and Treatment Exposure

**Figure 1:**
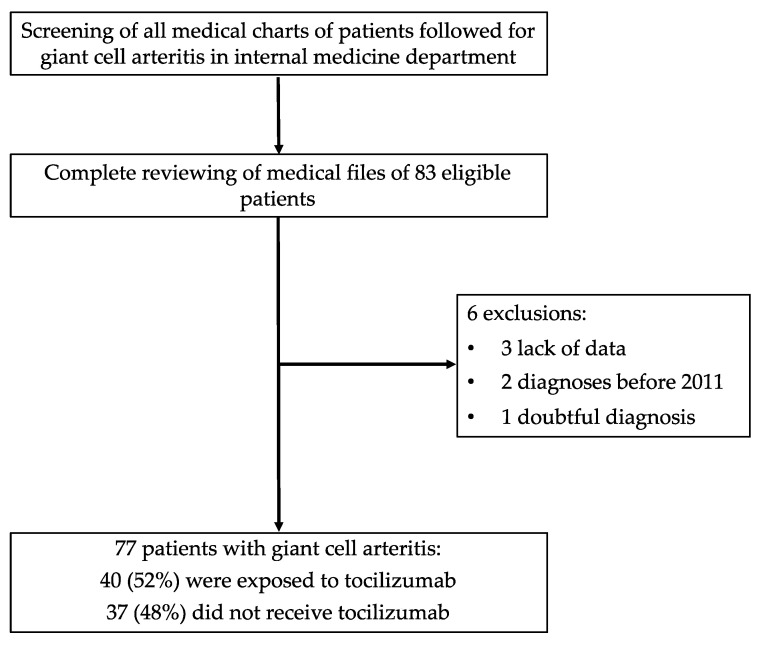
Flow chart of the selection of patients with giant cell arteritis followed at the Nimes University hospital.

Medical charts of the 83 potentially eligible patients were screened and six were excluded. Finally, we evaluated 77 GCA patients (Figure 1), including 48 (62%) women, mean age of 72.2 ± 9.8 years (median, 75 [64–79]). The population characteristics are summarized in Table 1. At baseline, 20 (26%) patients were overweight, 37 (48%) had hypertension, 17 (22%) were smokers, and 9 (12%) had diabetes mellitus. Twenty-six patients (34%) exhibited ophthalmological symptoms. The ACR and/or GiACTA criteria were fulfilled in 95% of the patients (other diagnoses were confirmed after a prolonged follow-up). The mean duration of the follow-up was 47.5 ± 28.8 months.

Fifty patients had a TAB; they did not differ from those without a TAB for baseline characteristics (age, sex, overweight, hypertension, diabetes, smoking, or dyslipidemia), but they exhibited more cephalic and ophthalmological symptoms. The patients with positive TAB (34/50) were older (76.0 ± 8.3 versus 68.2 ± 10.3 years old, *p* = 0.005), with a lower CRP level (88.3 ± 55.2 versus 168.1 ± 130.0 mg/L, *p* = 0.005) and more ophthalmological complications (53% versus 19%, *p* = 0.022) in comparison with negative TAB patients.

All the patients were treated with GCs (Table 1). The mean daily GCs dose over the treatment duration was 15.4 ± 7.3 mg/day (median, 13.9 [11.6–18.3] mg/day). Eighteen (23%) patients had a major GCs exposure, after a mean period of 18.7 ± 8.2 months after diagnosis. The mean duration of GCs treatment was 21.0 months (±20.5), and the mean cumulative GCs dose was 7977 mg (±4585) (median, 6900 [4950–9105] mg).

TCZ was used in 40 (52%) patients after a mean period of 9.5 ± 14.6 months post-diagnosis. TCZ was used for a mean period of 14.9 ± 7.9 months. Sixteen (40%) of these were early-treated patients without prior flare. TCZ was used in 27 (67.5%) patients for GCs dependence, in five (12.5%) cases for GC-related adverse events (mainly neuropsychiatric and metabolic effects), in five (12.5%) cases for both conditions, and in three (7.5%) cases for GCs resistance (two—with cephalic signs, one—with transient visual signs; all three exhibited a biological inflammatory syndrome despite a high dose of GCs). TCZ was administered by infusions of 8 mg/kg monthly, with a subcutaneous relay in three patients.

Among the 16 early-treated TCZ patients, the cumulative prednisone dose was lower (4295 ± 1613 versus 9307 ± 3948 mg; *p* < 0.01), and the duration of the GCs treatment was shorter (10 ± 3.7 versus 21.7 ± 12.2 months; *p* < 0.01) than in the 24 patients taking TCZ later. Among the 40 patients who received TCZ, seven (17.5%) had a major exposure to GCs. Details concerning TCZ safety are listed in Supplementary Table S1.

In the survival analysis, the patients with cephalic symptoms at baseline were less likely to receive TCZ, while the patients with the CRP level above 100 mg/L were at an increased risk of major GCs exposure (Supplementary Figure S1).

### 3.2. Outcomes

Among the 77 GCA patients, 59 (77%) reported at least one severe outcome. The types and mean delay of occurrence are detailed in Table 2. The median time for the first occurrence of a severe outcome was 13 [4–35] months.

**Figure 2:**
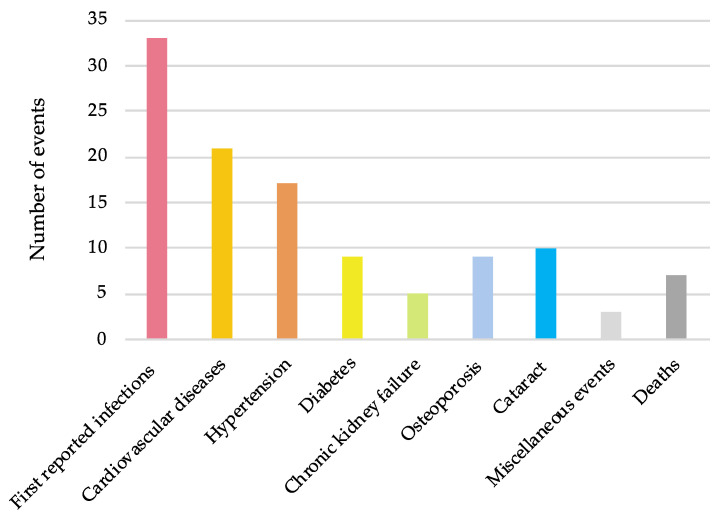
Repartition of the subgroups of the 114 severe outcomes recorded in the 77 patients with giant cell arteritis after a prolonged follow-up. Cardiovascular diseases combine stroke, myocardial infarction, acute heart failure, aneurysm, and peripheral arterial disease. For hypertension and diabetes, new-onset or worsening of previous corresponding conditions are reported. Miscellaneous events encompass neuropsychiatric severe symptoms and gastrointestinal hemorrhage.

We recorded 114 severe outcomes (including seven deaths). The subtype distribution is shown in Figure 2. The most common severe outcomes were infections, followed by cardiovascular diseases (20% of patients), cardiovascular risk factors (new-onset of worsening hypertension, and diabetes), fractural osteoporosis, and cataracts. We recorded two cases of severe neuropsychiatric symptoms (one delirium and one delusion) and one case of gastrointestinal hemorrhage (resulting in death).

We recorded three aneurysms: one concerned a smoking male patient with thoracoabdominal aortic aneurysm (78 mm) and subclavian aneurysm (36 mm), both treated by open surgery and prosthetic graft. At diagnosis, these large vessels showed an increased uptake of ^18^fluorodesoxyglucose. Among the five patients with strokes, two patients showed an increased uptake of ^18^fluorodesoxyglucose on supra-aortic trunks and/or vertebral arteries at GCA diagnosis.

**Figure 3:**
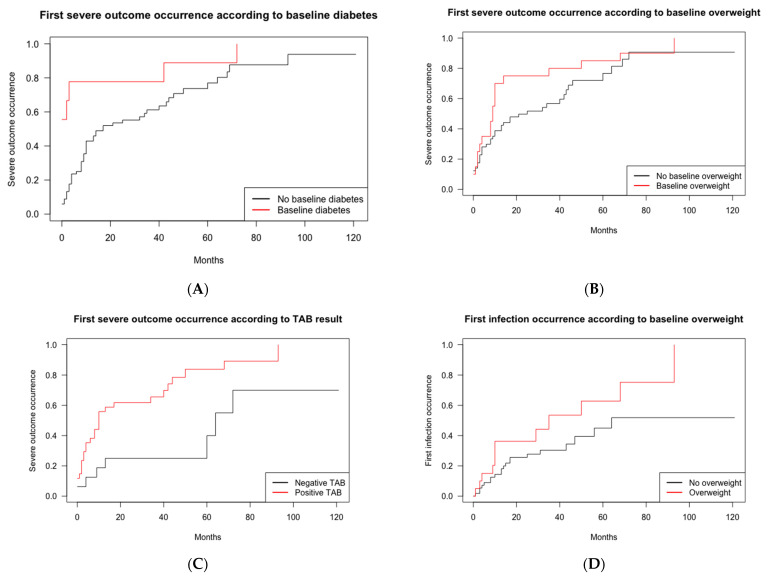
Risk of severe outcomes occurrence in the 77 patients with giant cell arteritis, according to the baseline characteristics (survival analysis). Survival curves illustrating severe outcomes onset according to the baseline characteristics in GCA patients. GCA, giant cell arteritis; TAB, temporal artery biopsy. The patients with baseline diabetes were at an increased risk of severe outcome onset (HR, 3.34 (1.52–7.35), *p* = 0.003) (**A**), as were the patients with baseline overweight (BMI ≥ 25 mg/m^2^) (HR, 2.06 (1.10–3.84), *p* = 0.023) (**B**), independently of age, sex, and baseline hypertension. (**C**) Among the patients with a TAB (*n* = 50), the patients with TAB positivity (*n* = 34) were at an increased risk of severe outcomes compared to the GCA patients with a negative TAB (HR, 5.34 (1.81–15.76), *p* = 0.002) independently of age, sex, overweight, and dyslipidemia (the association with diabetes and smoking was maintained). (**D**) The risk for the first infection was increased in the overweight GCA patients at baseline (HR, 2.47 (1.13–5.43), *p* = 0.024) independently of age, sex, diabetes, dyslipidemia, smoking, and major GCs exposure and TCZ exposure as time-dependent covariables.

The risk of occurrence of the first severe outcome in the survival analysis was increased in patients with diabetes at baseline (HR, 3.34 [1.52–7.35], *p* = 0.003) and in overweight patients (HR, 2.06 [1.10–3.84], *p* = 0.023) independently of age, sex, and hypertension (interaction between diabetes and overweight was observed). There was also a similar trend in smokers (HR, 2.23 [1.09–4.59], *p* = 0.028) and in patients with baseline dyslipidemia (HR, 2.33 [1.09–5.00], *p* = 0.030) (Figure 3). When TCZ and major GCs exposures were added as time-dependent covariates in the model, they were not positively correlated with the occurrence of severe outcomes, and the associations with baseline diabetes, overweight, and dyslipidemia were maintained (HR, 2.41 [1.05–5.55], *p* = 0.039; HR, 2.08 [1.14–3.81], *p* = 0.018; and HR, 2.27 [1.05–4.91], *p* = 0.038, respectively). Other baseline characteristics (e.g., cephalic or ophthalmological symptoms or the CRP level) were not associated with the incidence of the first severe outcome. When studying the first occurrence of strokes, myocardial infarctions, aneurysms, heart failure hospitalizations, peripheral arterial diseases, infections in hospitalizations, and deaths (*n* = 26 patients), TCZ and major GCs exposures (time-dependent covariates) were still not associated with the incidence of these outcomes (independently of age and sex).

In the subgroup of patients with a TAB (*n* = 50), patients with a positive TAB were at an increased risk of severe outcomes (HR, 5.34 [1.81–15.76], *p* = 0.002) independently of age, sex, overweight, and dyslipidemia (association with diabetes and smoking was maintained) (Figure 3). In the subgroup of patients who received TCZ, the number of severe outcomes did not differ between the early-treated patients and the other patients on TCZ (1.18 ± 0.88 versus 1.70 ± 1.43, *p* = 0.194).

Of the 33 first infections reported, five (15%) occurred after a major GCs exposure without TCZ, 10 (30%)—after a TCZ exposure (GCs < 10 g), and three (9%)—after both TCZ and major GCs exposure. Of the 57 total infections, 13 (23%) required hospitalization (two—in intensive care unit). The leading sites of infections were respiratory (37%) and urinary tract (37%), followed by skin (14%), digestive (3%), sepsis (2%), and miscellaneous infections (7%). We recorded two potentially opportunistic infections (one genital herpes, one zoster), and one SARS-CoV-2 infection. The incident risk of infection occurrence was increased only in the patients overweight at baseline (HR, 2.47 [1.13–5.43], *p* = 0.024) independently of age, sex, diabetes, dyslipidemia, smoking, major GCs exposure, and TCZ exposure as time-dependent covariables (Figure 3).

Seven (9%) patients died (five women and two men) during the follow-up at a mean age of 81.8 ± 7.6 years old: two—of cardiovascular events, one—of a severe infection, one—of gastrointestinal hemorrhage, three—of unknown causes. In the survival analysis, the risk of death was associated with the BMI value at diagnosis (19% increase for each point of the BMI, *p* = 0.034) independently of age.

All the patients were in complete remission during the first month after diagnosis, 96% in the third month, and 85% after one year. In the total study population, 48 (62%) patients experienced at least one relapse during the follow-up. The mean delay of the first relapse was 11.4 ± 6.8 months. The mean GCs dosage at the first relapse was 0.13 ± 0.15 mg/kg (prednisone-equivalent). The relapsing patients had a longer GCs treatment duration (25 ± 24 versus 14 ± 9 months; *p* < 0.001), and a higher cumulative GCs dose (7603 [5580–10,690] versus 5745 [4575–7110] mg; *p* = 0.007). Relapses occurred in 15 patients in the TCZ-exposed group, 12 of whom had stopped TCZ for a mean period of 6.1 ± 3.0 months, and three were currently on TCZ. In the early-treated TCZ-exposed patients, the number of relapses per patient was lower (0.59 versus 1.65; *p* = 0.0002) in comparison with the other patients in the TCZ-exposed group.

## 4. Discussion

In this study of 77 GCA patients, 77% of the patients experienced at least one severe outcome after a mean follow-up of 4 years. This proportion is consistent with previous studies [13,17,25]. GC-related adverse effects, such as new-onset or worsening of hypertension and diabetes, heart failure, infections, osteoporosis, and cataracts were the main causes of severe outcomes, as previously reported [7,13].

Our results highlighted a subcategory of GCA patients at high risk for severe outcomes: overweight patients, patients with diabetes or dyslipidemia, smokers, and patients with TAB positivity.

Diabetes has previously been associated with increased adverse events [13] and death from severe infections [26] in GCA patients. Interestingly, we also demonstrate that BMI/overweight was associated with severe outcomes, infection, and mortality occurrence in GCA patients. Being overweight may modulate GCs bioavailability, with overweight shown to be associated with an increased cumulative dose of GCs and higher plasma GCs concentration [27]. This hypothesis warrants further investigations. Overweight/obesity has been associated with an increased infection risk [28]. Moreover, we suspect an interaction between overweight and diabetes. To our knowledge, screening for overweight, rather than obesity, as a potential clinical predictive factor of severe outcomes onset, has never been considered before in GCA patients. However, because of the complex interplay between nutritional status, sarcopenia, fall risk, and loss of autonomy among these older patients, overweight should be seen as a frailty marker for severe outcomes onset but not a target-to-treat within this population.

We could not confirm the impact of age on severe outcomes occurrence in our survival analysis [6,11], probably because most patients fell into the same age range. However, we found that the patients with a positive TAB had a higher risk of severe outcomes. This may be supported by a patient- and disease-specific pattern. First, the patients with a TAB exhibited more cephalic and ophthalmological symptoms. Second, the TAB-positive patients were older, with a “cranial form” of the disease (cephalic and ophthalmological symptoms) and a lower CRP level, as opposed to a “systemic form” (or large-vessel vasculitis form) with constitutional signs, polymyalgia rheumatica, and cough [24,29,30]. Further studies focusing on this specific subgroup of patients and their comorbidities may provide insight into the underlying pathophysiological mechanisms.

Surprisingly, major cumulative GCs exposure did not affect the occurrence of severe outcomes. This contrasts with several published articles describing severe outcomes (and especially GC-related adverse effects such as cardiovascular diseases and infections) related to daily and cumulative GCs doses [4,5,6,7,31]. Some discrepancies can come from our methodology since we included cumulative GCs exposure as a time-dependent variable to study its impact on severe outcomes [7]. The observed cumulative GCs dose is consistent with previous real-world studies [6,23,24].

In these real-world patients, we did not find a positive association between TCZ exposure and severe outcomes independently of major GC exposure [32]. This is reassuring considering the expected increase in the plasma low-density lipoprotein (LDL) level on TCZ [33]. This may be partly explained by a broader vascular and metabolic protective effect of TCZ: decreased lipoprotein A and serum amyloid A high-density lipoprotein [34]; decreased insulin resistance through IL-17 inhibition [35]; decreased osteoclastic bone destruction [34]; and improved control of disease activity. Of note, therapeutic strategies targeting inflammation, including IL-6 blockers, are being studied for their potential role in avoiding atherosclerosis [36]. Our study demonstrated no decrease in incidental risk of severe outcomes in TCZ-exposed patients, even within the subgroup of early-treated patients. Our results are therefore consistent with the real-world study by Unizony et al., where the incidence of GC-related adverse events did not change with TCZ exposure [12].

To summarize, our findings suggest that patients and disease characteristics, especially traditional cardiovascular risk factors, but not treatment strategies, have a profound impact on severe outcomes onset and mortality. These risk factors could be evaluated through composite scores such as Charlson’s comorbidity index [37]. Interestingly, similar results have been reported in antineutrophil cytoplasmic autoantibody-associated vasculitis: the occurrence of major adverse cardiac events is associated with sedentary lifestyle, hypertension, dyslipidemia, and the number of cardiovascular risk factors, but not with the use of GCs or cyclophosphamide [38].

Considering the type of observed severe outcomes, the mean delay of occurrence, and risk factors, we suggest simple practical recommendations for this fragile population of GCA patients. First, it seems important to screen and treat new-onset hypertension and diabetes, especially within the first weeks after the diagnosis of GCA. They may be responsible for new hospitalizations and may later be complicated by cardiovascular diseases. We reported three cases of aneurysms associated with a long duration of treatment (≥18 months), and with previous increased ^18^fluorodesoxyglucose uptake on targeted vessels at GCA diagnosis in one case [39]. Second, as broadly recommended in this aging population, a careful use of potential nephrotoxic medications along with a tight control of diabetes and hypertension worsening may prevent the exacerbation of kidney injuries. Third, as respiratory infections are frequent in GCA patients, education about influenza and pneumococcal vaccination would be useful [13,40].

Finally, our results highlight the urgent need for screening and treating of fractural osteoporosis [13], in parallel with comprehensive fall risk prevention (including rehabilitation). Treatment of accelerated cataracts may be one such measure in this aging population, potentially contributing to a reduction in falls and morbidity.

Although we did not find an increased risk of severe outcomes occurrence after major GCs exposure in our study, literature data highlight that GCs exposure is the only modifiable factor associated with the development of GC-related adverse events [4,5,6,31]. Therefore, decreasing, rather than stopping, the cumulative dose of GCs appears to be critical in GCA patients. We described that TCZ was not associated with a decreased risk of severe outcomes in GCA patients. However, TCZ was introduced following current recommendations for its use in GCA patients (second-line treatment) [41]. The impact of TCZ exposure on severe outcomes may change if treatment strategies evolve towards earlier use of TCZ [10]. TCZ could thus be used as a GC-sparing agent. In the SEMIRA study in rheumatoid arthritis patients [42], prolonged use of low-dose steroids in addition to TCZ was associated with flare-free disease control, and fewer side effects. Another option could be to tailor the GCs tapering according to initial response, without further associated treatment. This stratified approach appeared to be effective in 73 GCA patients [43].

These tailored strategies should be first targeted to at-risk patients: overweight/diabetic patients with dyslipidemia and smokers, as well as positive-TAB patients. Comprehensive screening and follow-up of malnutrition, sarcopenia, fall-risk, along with preventive measures (vaccination, rehabilitation, decreasing polypharmacy), are mandatory in this frail population.

Strengths and limits: As this was a single-center retrospective study, we assume that most severe outcomes were recorded, and that few patients were lost to follow-up. This was a real-world study, including a wide spectrum of patients, with prolonged follow-up, allowing for long-exposure of GC-related adverse events (osteoporosis, cataract, atherosclerosis) to occur. Limitations mainly relate to ambulatory events (non-severe infections) that may have been poorly collected. We did not record less severe GC-related adverse events such as insomnia, weight gain, dyspepsia, myopathy or skin lesions, as reported in some studies [7,23]. Another limitation is the retrospective nature of the study: further prospective studies with random assignment of tocilizumab, and long-term follow-up, are necessary to address the potential impact of this treatment on severe outcomes onset.

## 5. Conclusions

Our study confirms that, in real-world GCA patients, outcomes including GC-related adverse events and geriatric morbidity are common, especially hypertension, diabetes, cardiovascular events, infections, and fractural osteoporosis. TCZ exposure does not seem to lower the occurrence of these outcomes. However, overweight, diabetic, dyslipidemic and smoking patients, as well as TAB-positive patients, are at increased risk of severe outcomes. Individual follow-up of these selected patients could be offered in daily practice, to reduce the burden of these comorbidities and associated hospitalizations. New therapeutic strategies aimed at lowering the cumulative dose of GCs should be considered, along with classical preventive measures involving frailty screening and prevention, impacting fall-risk and loss of autonomy in this aging population.

## Figures and Tables

**Table 1:** Baseline characteristics and treatment strategy in 77 patients with giant cell arteritis according to the severe outcome onset.

Baseline Characteristics	Total	GCA Patients with ≥1 Severe Outcome	GCA Patients without a Severe Outcome	*p*
	(*n* = 77)	(*n* = 59)	(*n* = 18)	
**Demographics**				
Age (mean ± SD) (years)	72.2 ± 9.8	72.5 ± 9.1	71.1 ± 11.9	0.60 ^#^
Sex (*n*, %) (female)	48 (62.3%)	39 (66.1%)	9 (50.0%)	0.22
**Past medical history**				
BMI (mean ± SD) (kg/m^2^)	23.9 ± 3.8	24.0 ± 4.1	23.6 ± 2.3	0.70
Overweight (BMI ≥ 25 kg/m^2^)	20 (25.9%)	19 (32.2%)	1 (5.6%)	**0.02**
Hypertension (*n*, %)	37 (48.1%)	30 (50.8%)	7 (38.9%)	0.37
Diabetes mellitus (*n*, %)	9 (11.7%)	9 (15.3%)	0 (0%)	0.08
Dyslipidemia (*n*, %)	12 (15.6%)	9 (15.3%)	3 (16.7%)	1.00 *
Smoking (*n*, %)	17 (22.1%)	13 (22.0%)	4 (22.2%)	1.00 *
Stroke (*n*, %)	3 (3.9%)	3 (5.1%)	0 (0%)	1.00 *
Coronary disease (*n*, %)	3 (3.9%)	3 (5.1%)	0 (0%)	1.00 *
Chronic kidney failure (*n*, %)	4 (5.2%)	4 (6.8%)	0 (0%)	0.57 *
Infection requiring hospitalization (*n*, %)	6 (7.8%)	5 (8.5%)	1 (5.6%)	1.00 *
Autoimmune diseases (*n*, %)	12 (15.6%)	11 (18.6%)	1 (5.6%)	0.27 *
**Treatment before diagnosis**				
Antiplatelet agents (*n*, %)	14 (18.2%)	13 (22.9%)	1 (5.6%)	0.17 *
Anticoagulation (*n*, %)	4 (5.2%)	3 (5.1%)	1 (5.6%)	1.00 *
Dyslipidemia treatment (*n*, %)	9 (11.7%)	8 (13.6%)	1 (5.6%)	0.68 *
Immunosuppressive agents (*n*, %)	3 (3.9%)	3 (5.1%)	0 (0%)	1.00 *
**Clinical GCA manifestations**				
Cephalic symptoms (*n*, %) ^¶^	61 (79.2%)	45 (76.3%)	16 (88.9%)	0.33 *
Constitutional symptoms (*n*, %) ^µ^	54 (70.1%)	40 (67.8%)	14 (77.8%)	0.42
Polymyalgia rheumatica (*n*, %)	19 (24.7%)	15 (25.4%)	4 (22.2%)	1.00 *
Coughing (*n*, %)	12 (15.6%)	11 (18.6%)	1 (5.6%)	0.27
Ophthalmological symptoms (*n*, %) ^‖^	26 (33.8%)	20 (33.9%)	6 (33.3%)	0.96
**Laboratory tests and biopsy**				
C-reactive protein (median (IQR)) (mg/L)	100 [58–135]	98 [54–136]	100 [72–125]	0.64
Positive temporal artery biopsy (*n*, %)	34/50 (68%)	28/35 (80%)	6/15 (40%)	**<0.01**
**GCs treatment strategy**				
GCs pulse therapy (*n*, %)	11 (14.3%)	9 (15.3%)	2 (11.1%)	1.00 *
GCs duration (median (IQR)) (months)	16 [12–20]	16 [12–20]	14 [11–20]	0.79
Cumulative dose (mean ± SD) (mg)	7977 ± 4585	7852 ± 4059	8388 ± 6129	0.86
Mean GCs dose/day over the GCs treatment period (mean ± SD) (mg)	15.4 ± 7.3	15.6 ± 8.2	14.7 ± 3.4	0.64
Major exposure to GCs (*n*, %) ^α^	18 (23.4%)	15 (25.4%)	3 (16.7%)	0.54 *
Patients under GCs at the end of the follow-up (*n*, %)	17 (24.3%)	10 (16.9%)	7 (38.9%)	0.12 *
**TCZ treatment**				
TCZ exposure	40 (51.9%)	34 (57.6%)	6 (33.3%)	0.07
Time until TCZ introduction (mean ± SD) (months)	9.5 ± 14.6	9.7 ± 15.6	8.5 ± 7.1	0.86
**Associated treatment at** **diagnosis**				
Aspirin (*n*, %)	30/63 (47.6%)	24/46 (52.2%)	6/17 (35.3%)	0.23
Osteoporosis prevention (*n*, %) ^†^	57 (75%)	41 (69.5%)	16 (94.1%)	0.05 *
Vaccinations up to date (*n*, %) ^£^	10 (13%)	7 (11.9%)	3 (16.7%)	0.69
**Follow-up (mean ± SD) (months)**	47.5 ± 28.8	49.1 ± 29.4	41.3 ± 26.9	0.32

GCA: giant cell arteritis; SD, standard deviation; TCZ: tocilizumab; BMI: body mass index. All the quantitative statistical comparisons were performed with the Wilcoxon–Mann–Whitney rank sum test, except those with a ^#^, performed with Student’s test. All the qualitative statistical comparisons were performed with the chi^2^ test, except those with a *, performed with Fisher’s test. Among the past autoimmune diseases, we recorded six patients with previous polymyalgia rheumatica, two—with thyroiditis, one—with rheumatoid arthritis, one—with psoriasis, one—with scleroderma of Buschke, one—with GCA (in remission for 10 years). ^¶^ Cephalic symptoms were headache, scalp tenderness, or jaw claudication. ^µ^ Constitutional symptoms concerned fever, asthenia, anorexia, or weight loss. ^‖^ Ophthalmological symptoms were transient or permanent vision loss, allegedly due to GCA. ^α^ Major exposure to GCs therapy was defined by a cumulative dose > 10 g (prednisone-equivalent). ^†^ Osteoporosis prevention was defined by the prescription of calcium, vitamin D, and bisphosphonates. ^£^ Vaccinations up to date were defined by an update of influenza or pneumococcal vaccines.

**Table 2:** Description of 114 severe outcomes and their mean delay of occurrence in the 77 patients with giant cell arteritis.

Types of Outcomes	Number of Recorded Outcomes (*n*, % of Total Patients)	Median Delay of Occurrence (Months) (IQR) after GCA Diagnosis
Stroke	5 (6.5%)	9 [9–42]
Myocardial infarction	1 (1.3%)	93 [-]
Acute heart failure	8 (10.4%)	8 [5.3–30.3]
Aneurysm	3 (3.9%)	28 [21–54]
Peripheral arterial disease	4 (5.2%)	20 [10–43]
Hypertension	17 (22.1%)	9 [0–42]
Diabetes	9 (11.7%)	0 [0–1]
Chronic kidney failure	5 (6.8%)	26 [11–45]
First reported infection	33 (42.9%)	13 [7–32]
Osteoporosis	9 (11.7%)	14 [8–34]
Cataract	10 (13%)	13 [4–32]
Severe neuropsychiatric event	2 (2.6%)	2 [-]
Gastrointestinal hemorrhage	1 (1.3%)	40 [-]
Death	7 (9.1%)	63 [28–85]

## Data Availability

Anonymized data access is available on request to the corresponding author.

## Supplementary Materials

The following supporting information can be downloaded at: https://www.mdpi.com/article/10.3390/jcm11113115/s1, Figure S1: Risk of treatment exposures in real-world giant cell arteritis patients, according to baseline characteristics, Table S1: Suspected tocilizumab-related effects, and tocilizumab treatment strategy, in patients with giant cell arteritis under tocilizumab.
