# Supplementary material for: Patients’ Baseline Characteristics, but Not Tocilizumab Exposure, Affect Severe Outcomes Onset in Giant Cell Arteritis: A Real-World Study
Source: J Clin Med. 2022 May 31;11(11):3115. doi: 10.3390/jcm11113115 (PMC9181652; doi:10.3390/jcm11113115)
Supplement: Supplementary file 1 [file jcm-11-03115-s001.zip › jcm-1710603-supplementary.pdf]

Journal of
Clinical Medicine

MDPI

Supplementary Table S1. Suspected tocilizumab-related effects, and tocilizumab treatment strategy, in patients with giant cell arteritis under tocilizumab.

Suspected tocilizumab-related effects	Total (n=40)
At least one infection (n, %)	13 (32.5%)
Dyslipidemia (n, %) ¶	5/14 (35.7%)
Hepatic cytolysis (n, %) µ	4 (10.0%)
Neutropenia (n, %) ǁ	2 (5.0%)
Digestive or hypersensitivity reaction (n, %)	0 (0%)
**Treatment strategy**	
Discontinuation (n, %)	0 (0%)
Duration of use (mean ±SD) (months)	14.9±7.9
Early-treated patients * (n, %)	16 (40%)
Prolonged use of TCZ ≥ 12 months (n, %)	14 (35.0%)
Prolonged use of TCZ ≥ 18 months (n, %)	9 (22.5%)

TCZ: tocilizumab.
¶ Dyslipidemia was defined by a LDL level increase > 50% after tocilizumab exposure.
µ Hepatic cytolysis was defined by an ALT/AST level > 1.5N after tocilizumab exposure.
ǁ Neutropenia was defined by a count of neutrophils <1G/L after tocilizumab exposure.
* Early-treated patients received TCZ within 3 months after the diagnosis of giant cell arteritis.

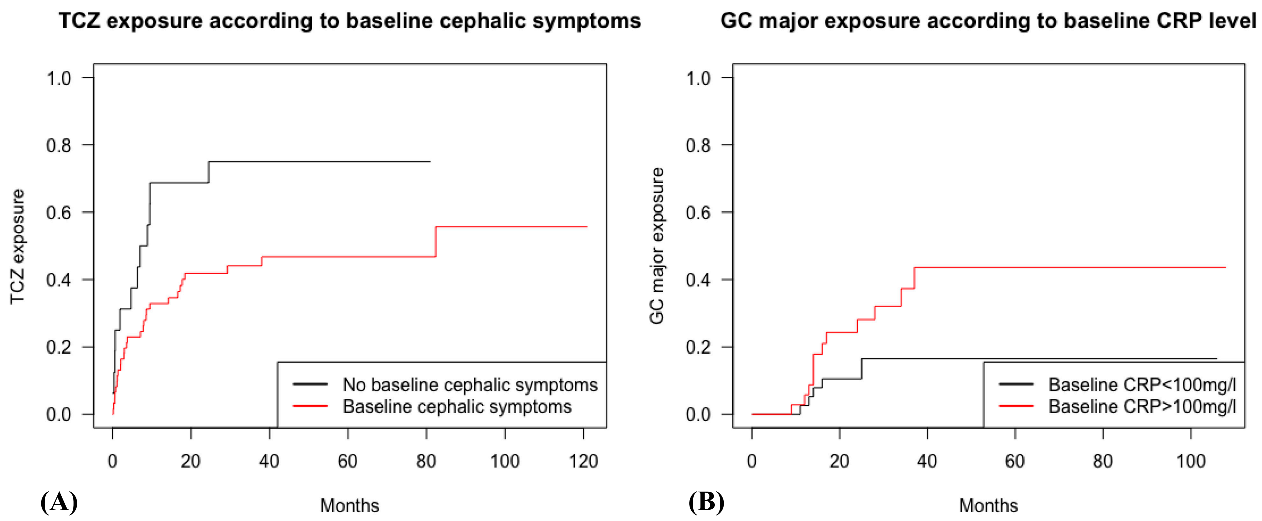

Supplementary Figure S1. Risk of treatment exposures in "real-world" giant cell arteritis patients, according to baseline characteristics. TCZ, Tocilizumab; GC, Glucocorticoids; CRP, C-reactive protein; GCA, giant cell arteritis. Survival curves illustrating treatment exposure risk according to baseline characteristics in GCA patients. (**A**) GCA patients with cephalic symptoms (headache, scalp tenderness or jaw claudication) at baseline were less likely to receive TCZ (HR 0.29, 95% CI [0.12-0.72], p=0.008), independently of age, sex, diabetes, overweight (BMI ≥25mg/m²), smoking, baseline ophthalmological symptoms and CRP level. (**B**) GCA patients with baseline CRP level above 100mg/l were at increased risk of GC major exposure, defined by a cumulative dose >10 grams of equivalent-prednisone (HR 3.08 [1.02-9.34], p=0.047), independently of age, sex, diabetes, overweight, smoking, baseline ophthalmological and cephalic symptoms.
